# Psycho-socio-economic factors and risk of cardiorenal multimorbidity in middle to older-aged adults: prospective findings from the Canadian longitudinal study on aging

**DOI:** 10.1007/s11357-025-02081-y

**Published:** 2026-01-05

**Authors:** Setor K. Kunutsor, Reyhaneh Rikhtehgaran, Anita Soni

**Affiliations:** 1 Section of Cardiology, Department of Internal Medicine, Max Rady College of Medicine, Rady Faculty of Health Sciences, University of Manitoba, 409 Tache Avenue, St. Boniface Hospital, Winnipeg, MB R2H 2A6 Canada; 2 Cardiovascular Real-World Evidence (CARDIO-RWE) Research Group, Section of Cardiology, I.H. Asper Institute, St. Boniface Hospital, 369 Tache Avenue, Winnipeg, MB R2H 2A6 Canada; 3 Department of Physiology and Pathophysiology, Rady Faculty of Health Sciences, University of Manitoba, Manitoba, Canada

**Keywords:** Psycho-socio-economic factor, Cardiorenal multimorbidity, Prospective study, CLSA

## Abstract

Psycho-socio-economic factors (PSEFs) such as income and homeownership may influence the prevalence of cardiorenal multimorbidity (CRM), yet their prospective associations with CRM risk remain unclear. This study aimed to estimate CRM incidence and examine its relationships with multiple PSEFs in a nationally representative Canadian cohort. We analyzed data from 16,557 participants (mean age: 60.4 years; 48.9% men) in the Canadian Longitudinal Study on Aging (CLSA) who were free of CRM at baseline (2010–2015). Incident CRM was defined as the co-occurrence of at least one cardiovascular disease and kidney disease at second follow-up (2018–2021). Survey-weighted multivariable logistic regression estimated odds ratios (ORs) and 95% confidence intervals (CIs) for PSEFs and CRM risk. Over 3–11 years follow-up, 194 participants (1.2%) developed CRM, corresponding to 8.80 per 1,000 individuals (95% CI: 6.77–11.40). CRM incidence increased with age, was higher in men than women (10.63 vs. 7.35 per 1,000), urban than rural residents (9.69 vs. 3.72 per 1,000), and immigrants than non-immigrants (10.87 vs. 8.49 per 1,000). Greater tangible social support reduced CRM risk (OR = 0.65; 95% CI: 0.43–0.97), while depression (OR = 1.68; 95% CI: 1.07–2.65) and PTSD (OR = 3.14; 95% CI: 1.74–5.67) increased risk. In middle- to older-aged Canadian adults, higher social support appears protective, whereas depression and PTSD increase CRM risk. Although incidence was low, CRM burden rose with age and was higher among men, urban residents and immigrants. These findings highlight the importance of psychosocial well-being and social connectedness in preventing CRM and reducing multimorbidity burden in aging populations.

**Supplementary Information:**

The online version contains supplementary material available at https://doi.org/10.1007/s11357-025-02081-y.

## Introduction

Cardiovascular disease (CVD) and chronic kidney disease (CKD) are leading contributors to morbidity, mortality, and healthcare expenditures in Canada. CVD accounts for nearly one in four deaths nationally [1], while CKD affects an estimated 12% of Canadians [2], with both conditions disproportionately impacting older adults and individuals of lower socioeconomic status [3]. These chronic conditions often share overlapping risk factors, pathophysiological mechanisms, and complications, leading to their frequent co-occurrence—a clinical construct increasingly referred to as cardiorenal multimorbidity (CRM) [4, 5]. CRM, characterized by the coexistence of CVD and CKD within an individual, poses unique clinical challenges, including complex care needs, accelerated disease progression, and elevated risk of hospitalization and premature mortality [6, 7]. Despite its potential significant health burden, CRM has historically received limited attention in population health research, particularly in relation to its social determinants. Using data from the Canadian Longitudinal Study on Aging (CLSA), our prior cross-sectional analysis provided one of the first national estimates of CRM prevalence in middle-aged and older adults [8]. We reported that CRM, though relatively infrequent, exhibited clear sociodemographic gradients—being more common in older individuals, males, and those residing in rural areas of Canada [8].

Growing evidence highlights the role of psycho-socio-economic factors (PSEFs), including household income, educational attainment, employment status, housing stability, social support, and marital status, as important determinants of chronic disease risk [9–12]. These upstream exposures shape access to healthcare, health behaviors, stress resilience, and overall life opportunities, and have been independently associated with the development of CVD and CKD [13–18]. However, little is known about their influence on CRM as a distinct multimorbidity phenotype. In our previous work using cross-sectional CLSA data, we explored the associations between PSEFs and prevalent CRM and found that homeownership and, to a lesser extent, moderate income levels were linked to lower odds of CRM [8]. While informative, such analyses are inherently limited in their ability to disentangle temporality and directionality, raising concerns about reverse causation. For example, the onset of multimorbidity may influence an individual’s income or housing situation, rather than the other way around.

To address these limitations, we now leverage the longitudinal follow-up of the CLSA to investigate the prospective relationships between PSEFs and CRM. The CLSA provides a unique platform to evaluate the incidence of CRM over time in a large, nationally representative aging population, while accounting for individual sociodemographic, psychosocial, and geographic contexts.

This study aims to (i) estimate the incidence of CRM in the Canadian population, overall and stratified by age, sex, geographical location, and immigration status and (ii) assess the prospective associations between multiple PSEFs and risk of developing CRM.

## Materials and methods

### Study design and population

This study was designed and reported in accordance with the STROBE (Strengthening the Reporting of Observational Studies in Epidemiology) guidelines (**Supplementary Material 1**) and the Statistical Analyses and Methods in the Published Literature (SAMPL) recommendations [19]. It employed a prospective population-based design using baseline and second follow-up data from the CLSA, with data collection occurring between 2010–2015 and 2018–2021, respectively. The CLSA is a large, nationally representative cohort comprising over 50,000 individuals aged 45 to 85 at recruitment. Participants were enrolled through a combination of strategies, including the Canadian Community Health Survey on Healthy Aging, provincial health insurance registries, and random-digit dialing [20]. The CLSA consists of two sub-cohorts: the Tracking Cohort (n = 21,241), which was assessed via 60-min telephone interviews, and the Comprehensive Cohort (n = 30,097), which underwent in-depth in-person assessments, physical measurements, and biological sampling either at data collection centres or in-home visits [21, 22].

For this analysis, we restricted our sample to individuals in the Comprehensive Cohort to leverage the rich dataset on clinical, behavioral, psychosocial, and biochemical factors, including lipid markers. Participants were selected from within 25–50 km of 11 collection centres across Canada using random sampling methods. The CLSA excluded individuals residing in the three territories (Yukon, Northwest Territories, and Nunavut), people living on federal First Nations reserves, full-time Canadian Armed Forces members, and those unable to communicate in English or French. Individuals living in institutions and those with significant cognitive impairment were excluded at baseline. In addition, participants who identified with a gender other than male or female were excluded from our analysis due to limited sample size. We further excluded participants with prevalent CRM at baseline. The final analytical sample included 16,557 individuals with complete data on PSEFs, covariates, CRM status at baseline and incident CRM status. Ethical approval for the CLSA was obtained from 13 institutional review boards across Canada. Written informed consent was obtained from all participants. Ethical approval for the present secondary analysis was provided by the University of Manitoba Health Research Ethics Board (HS26767).

### Assessment of PSEFs and risk markers

We examined several demographic and socio-economic variables in the analysis. Age, biological sex, immigration status, and place of residence (urban vs. rural) were included, with geographic classification determined using postal codes as previously described [23]. Socioeconomic status was assessed through indicators such as total household income, highest level of education attained, homeownership (classified as "own" for homeowners and "other" for all other arrangements), marital status (categorized as married/common-law or single/previously married, including separated, divorced, or widowed), and current employment status (dichotomized into “current worker” and “non-current worker”). Missing data on employment status were supplemented using responses on retirement and work hours: participants who reported being fully retired and had no work hours were considered non-current workers, while those who reported any working hours or were partly retired were classified as current workers.

Psychosocial variables included multiple dimensions of social support, social participation, satisfaction with life, loneliness, social network size, and the presence of depressive symptoms, anxiety, and post-traumatic stress disorder (PTSD). Social support was evaluated using the 19-item Medical Outcomes Study (MOS) Social Support Survey, which measures four domains—tangible support, emotional/informational support, affectionate support, and positive social interaction—combined into a functional social support index ranging from 0 to 100, with higher scores reflecting stronger perceived support [24]. Both overall and domain-specific scores were analyzed and categorized into tertiles, with the lowest tertile indicating low support and the combined middle and upper tertiles representing moderate to high support. Life satisfaction was assessed using the Satisfaction with Life Scale and categorized into two groups: “low” for the first tertile and “medium–high” for the combined middle and upper tertiles [25]. Social participation was calculated as a monthly activity frequency score across eight domains: social outings with family/friends, religious involvement, physical activity with others, educational or cultural activities, club or fraternal group involvement, association participation, volunteer work, and other recreational engagements. Frequency responses were converted to monthly activity counts, e.g., “daily” = 20, “weekly” = 6, “monthly” = 2, “yearly” = 1, and “never” = 0, and summed to derive a total participation score, with higher values indicating more frequent engagement [26]. Social network size was calculated as a continuous variable representing the total number of living children, siblings, close friends, and neighbors, with higher scores signifying larger social networks [27]. Depressive symptoms were assessed using the 10-item version of the Center for Epidemiologic Studies Depression Scale (CES-D-10) [28]. Loneliness was derived from the CES-D item on loneliness frequency and categorized as “rarely or never” (less than 1 day/week) or “felt lonely” (1 + days/week).

Behavioral variables included alcohol use, categorized as never, light/moderate (1–15 drinks/week for men; 1–10 for women), or heavy drinkers (≥ 15 drinks/week for men; ≥ 10 for women); and smoking status, grouped into non-smokers (never smoked or former occasional smokers) and smokers (current or former daily smokers, or current occasional smokers) [29]. Physical activity was assessed with the Physical Activity Scale for the Elderly (PASE), a validated tool capturing occupational, household, and leisure activities in older adults [30]. Walking frequency was recoded into three categories: inactive (0 days/week), insufficiently active (1–4 days/week), and sufficiently active (5–7 days/week), based on established research practices. Clinical assessments included anthropometric and biochemical measurements. Height and weight were measured using standardized protocols, and body mass index (BMI) was calculated as weight (kg) divided by height squared (m^2^). Blood samples were collected via non-fasting venipuncture and processed at Calgary Laboratory Services. Serum concentrations of total cholesterol, high-density lipoprotein cholesterol (HDL-C), and triglycerides were quantified using the Roche Diagnostics Cobas 8000 analyzer platform [31]. Self-reported chronic conditions expected to persist for at least six months were used to identify health conditions. Diabetes was defined as a physician-diagnosed history of diabetes, borderline diabetes, or high blood sugar, while hypertension was based on self-reported physician diagnosis.

### Ascertainment of outcome

Incident CRM was identified when a participant reported both a CVD and kidney disease at the second follow-up (2018–2021). CVDs included angina, myocardial infarction, peripheral vascular disease, stroke, and heart failure. CRM was treated as a dichotomous outcome variable, classified as either present or absent. Given the longitudinal nature of the CLSA and its aging population, inconsistencies in self-reported chronic conditions across study waves were not uncommon [32].  For example, participants sometimes answered “yes” to a diagnosis in one wave but “no” in a subsequent one, or provided a “not applicable” (NA) response that later changed to a definitive “yes” or “no.” To harmonize these discrepancies and ensure more accurate classification of CRM status, we applied a series of decision rules. If a response of “NA” was followed by “no,” it was interpreted as “no,” while an “NA” following a prior “yes” was treated as “yes.” In cases where two consecutive “NA” responses were later followed by “no,” all responses were coded as “no.” Conversely, if a “yes” was recorded at baseline and followed by two “NA” responses in subsequent waves, the diagnosis was preserved as “yes.” When participants gave inconsistent but complete responses across waves, we retained the earliest affirmative response, recognizing the possibility of recall issues, particularly over long follow-up intervals. Although this approach may slightly overestimate the incidence of CRM, it prioritizes consistency and minimizes under-ascertainment in the context of chronic disease reporting.

### Statistical analysis

All statistical procedures incorporated weights to improve the representativeness of the results for the Canadian population and to adjust for the CLSA’s complex sampling strategy. Descriptive analyses (excluding frequency counts) used inflation weights, whereas regression models employed analytic weights, as recommended in CLSA documentation [33]. We described baseline participant characteristics using summary statistics, expressing continuous variables as means with standard deviations (SDs) or medians with interquartile ranges (IQRs), based on distributional properties. Categorical data were summarized using counts and proportions. Between-group comparisons were performed using methods that accounted for the survey design: design-corrected t-tests for normally distributed continuous variables, non-parametric tests for skewed data, and Rao-Scott chi-square tests for categorical variables. Incidence rates of CRM per 1,000 individuals were calculated overall and stratified by sociodemographic categories. Associations between PSEFs and CRM were examined using multivariable logistic regression models with survey weights, reporting odds ratios (ORs) and 95% confidence intervals (CIs). Three sequential adjustment models were used: Model 1 adjusted for age and sex; Model 2 additionally adjusted for geographic region, immigration status, BMI, lipid levels (total and HDL-C), smoking, alcohol intake, hypertension, and diabetes; and Model 3 further adjusted for physical activity. Selection of covariates was guided by previous CLSA-based studies, [8, 34, 35] established links with cardiometabolic outcomes, and their potential role as confounders [36]. Analyses were conducted using R software (version 4.4.2; R Foundation for Statistical Computing, Vienna, Austria).

## Results

Among 16,557 participants (mean age 60.4 ± 9.0 years; 48.9% men), those who developed CRM were generally older, had lower household income, and were less likely to have post-secondary education, own a home, or be employed (Table 1). They also reported higher levels of loneliness, depression, anxiety, and PTSD, were more often physically inactive, and had greater prevalence of hypertension, diabetes or prediabetes, higher BMI, and lower HDL-C levels.

**Table 1:** Baseline characteristics of participants overall and by incident CRM status

Variable	Overall (*N* = 16557) Mean (SD) or median (IQR)	No CRM (*N* = 16363) Mean (SD) or median (IQR)	Incident CRM (*N* = 194) Mean (SD) or median (IQR)	*p*-value
Household Income, n (%)				< 0.001
< $50 K	3764 (25.3)	3693 (25.2)	71 (40.3)	
$50 K—99 K	6031 (35.8)	5963 (35.8)	68 (38.8)	
≥ $100 K	6762 (38.8)	6707 (39.0)	55 (20.8)	
Post-secondary education, n (%)	14478 (73.8)	14317 (73.8)	161 (69.1)	0.04
Homeowner, n (%)	14601 (86.4)	14456 (86.5)	145 (72.1)	0.001
Married/Common-law, n (%)	12028 (76.4)	11903 (76.5)	125 (72.9)	0.31
Current worker, n (%)	9177 (57.7)	9112 (58.0)	65 (32.8)	< 0.001
Social support	85.53 (73.68, 96.05)	85.53 (73.68, 96.05)	86.84 (69.74, 93.42)	0.82
Social participation	15.00 (10.00, 22.00)	15.00 (10.00, 22.00)	13.00 (9.00, 20.00)	0.08
Affection score	100.00 (83.33, 100.00)	100.00 (83.33, 100.00)	100.00 (75.00, 100.00)	0.88
Emotional & Informational Support	84.38 (68.75, 96.88)	84.38 (68.75, 96.88)	84.38 (71.88, 93.75)	0.90
Tangible Social Support	87.50 (75.00, 100.00)	87.50 (75.00, 100.00)	87.50 (62.5, 100.00)	0.57
Positive Social Interaction	91.67 (75.00, 100.00)	91.67 (75.00, 100.00)	91.67 (75.00, 100.00)	0.73
Satisfaction with life	30.00 (25.00, 33.00)	30.00 (25.00, 33.00)	30.00 (22.00, 33.00)	0.05
Loneliness, n (%)	3806 (24.1)	3749 (24.1)	57 (31.1)	0.01
Depression, n (%)	2146 (15.2)	2104 (15.0)	42 (27.3)	< 0.001
Anxiety, n (%)	1301 (8.3)	1278 (8.2)	23 (13.8)	0.02
PTSD, n (%)	708 (4.2)	687 (4.1)	21 (14.2)	< 0.001
Network size	17.00 (12.00, 24.00)	17.00 (12.00, 24.00)	15.00 (9.00,23.00)	0.27
Males, n (%)	8360 (48.9)	8248 (48.8)	112 (61.1)	0.08
Immigrants, n (%)	2739 (17.1)	2692 (17.0)	47 (22.2)	0.06
Age (years)	60.36 (8.96)	60.30 (8.93)	66.61 (9.67)	< 0.001
Age group				< 0.001
45–54	4497 (31.4)	4481 (31.6)	16 (13.8)	
55–64	6007 (37.4)	5959 (37.5)	48 (28.6)	
65–74	3992 (21.8)	3922 (21.7)	70 (30.0)	
75 +	2061 (9.4)	2001 (9.2)	60 (27.6)	
Urbanized area resident, n (%)	15085 (94.2)	14898 (94.1)	187 (97.2)	0.05
Smoker, n (%)	7980 (54.4)	7862 (54.4)	118 (54.1)	0.01
Alcohol, n (%)				0.06
Never drinker	3730 (21.8)	3677 (21.7)	53 (26.4)	
Light/Moderate drinker	10708 (65.0)	10584 (65.0)	124 (63.7)	
Heavy drinker	2119 (13.3)	2102 (13.3)	17 (9.9)	
Physical activity, n (%)				0.02
Inactive	2246 (13.2)	2204 (13.1)	42 (24.1)	
Insufficiently active	5593 (34.1)	5531 (34.2)	62 (27.9)	
Sufficiently active	8718 (52.7)	8628 (52.7)	90 (48.0)	
Diabetes diagnosis, n (%)	2556 (16.1)	2492 (15.9)	64 (33.3)	< 0.001
Hypertension, n (%)	5733 (33.3)	5607 (33.0)	126 (62.4)	< 0.001
BMI (kg/m^2^)	28.28 (5.42)	28.26 (5.40)	30.52 (6.96)	< 0.001
Total cholesterol (mmol/L)	5.20 (1.06)	5.20 (1.05)	4.77 (1.37)	< 0.001
HDL-C (mmol/L)	1.50 (0.47)	1.50 (0.47)	1.32 (0.42)	0.002

Continuous variables are reported as mean (SD) or median (interquartile range) and categorical variables are reported as n (%)

Abbreviations: BMI, body mass index; CRM, Cardiorenal Multimorbidity; HDL-C, high-density lipoprotein cholesterol; IQR, interquartile range; PTSD, post-traumatic stress disorder; SD, standard deviation

Never drinkers were those who reported no alcohol consumption; light/moderate drinkers were those who consumed 1–15 drinks per week for men and 1–10 drinks per week for women; heavy drinkers consumed more than 15 drinks per week for men and more than 10 drinks per week for women. Inactive individuals were those who reported never walking outside; insufficiently active individuals were those walking outside 1–4 days per week; sufficiently active individuals were those walking outside 5–7 days per week. Diabetes diagnosis indicates self-reported doctor diagnosis of diabetes, borderline diabetes, or high blood sugar

Descriptive statistics (except for frequencies) were calculated using inflation weights, while *p*-values were calculated using analytic weights from the second follow-up, as per the CLSA protocol, to ensure the results are more representative of the Canadian population

Over a 3–11 year follow-up, 194 participants (1.2%) developed CRM, corresponding to an adjusted incidence rate of 8.80 per 1,000 individuals (95% CI: 6.77–11.40) (Table 2). The incidence increased with age, reaching 27.51 per 1,000 (95% CI: 18.68–40.35) among adults aged ≥ 75 years. CRM incidence was higher in men than women (10.63 vs 7.35 per 1,000), in urban compared with rural residents (9.69 vs 3.72 per 1,000), and in immigrants compared with non-immigrants (10.87 vs. 8.49 per 1,000).

**Table 2:** Estimated incidence of CRM per 1,000 population, by age group, sex, location, and immigration status

	Incidence (95% CI)
Overall	8.80 (6.77, 11.40)
Age group	
45–54	4.19 (2.28, 7.67)
55–64	8.96 (6.17, 13.01)
65–74	15.42 (11.08, 21.42)
75 +	27.51 (18.68, 40.35)
Sex	
Men	10.63 (7.94, 14.20)
Women	7.35 (5.13, 10.50)
Location	
Urbanized Area	9.69 (7.51, 12.50)
Rural Area	3.72 (1.25, 11.00)
Immigration status	
Immigrant	10.87 (7.11, 16.60)
Non-immigrant	8.49 (6.43, 11.20)

*CI* confidence interval,* CRM* Cardiorenal Multimorbidity

The overall incidence estimate is adjusted for age group, sex, location and immigration status

The age group–stratified incidence is adjusted for sex, location and immigration status

The sex-stratified incidence is adjusted for age group, location and immigration status

The location-stratified incidence is adjusted for sex, age group and immigration status

The immigration-stratified incidence is adjusted for sex, age group and location

Analytic weights from the second follow-up are used in the calculation of estimates and confidence intervals

Odds ratios for the associations of PSEFs with the risk of incident CRM are reported in Table 3. In age- and sex-adjusted analyses, participants with high tangible social support had significantly lower odds of developing CRM compared to those with low support (OR = 0.67; 95% CI: 0.45–0.99). This association remained robust after further adjustment for lifestyle behaviours, lipid profile, and comorbidities (OR = 0.65; 95% CI: 0.43–0.97). Having depression (OR = 1.68; 95% CI: 1.07–2.65) and PTSD (OR = 3.14; 95% CI: 1.74–5.67) were each associated with an increased odd of developing CRM. Loneliness showed a modest but not statistically significant association with increased CRM risk (OR = 1.58; 95% CI: 0.97–2.56). No clear associations were observed between CRM and other PSEF factors including household income, education level, homeownership, marital status, employment status, satisfaction with life, social participation, and self-reported anxiety.

**Table 3:** Associations of PSEFs with the risk of incident CRM

PSEFs	Events/Total participants	Model 1		Model 2		Model 3	
		OR (95% CI)	*p*-value	OR (95% CI)	*p*-value	OR (95% CI)	*p*-value
Household Income							
< $50 K	71/3764	ref		ref		ref	
$50 K—99 K	68/6031	0.86 (0.50–1.46)	.57	0.87 (0.51–1.49)	.61	0.87 (0.51–1.49)	.62
≥ $100 K	55/6762	0.83 (0.47–1.47)	.53	0.89 (0.50–1.59)	.70	0.89 (0.50–1.60)	.71
Education							
No Post-secondary education	33/2079	ref		ref		ref	
Post-secondary education	161/14478	0.95 (0.58–1.56)	.84	1.04 (0.62–1.74)	.88	1.04 (0.62–1.74)	.88
Homeowner							
No	49/1956	ref		ref		ref	
Yes	145/14601	0.63 (0.40–1.00)	.05	0.71 (0.45–1.12)	.14	0.69 (0.44–1.08)	.11
Marital Status							
Single/Previously married	69/4529	ref		ref		ref	
Married/Common-law	125/12028	1.24 (0.75–2.03)	.40	1.21 (0.74–1.97)	.45	1.20 (0.74–1.95)	.47
Employment Status							
Non-current worker	129/7380	ref		ref		ref	
Current worker	65/9177	0.76 (0.44–1.33)	.34	0.80 (0.47–1.36)	.41	0.79 (0.46–1.36)	.40
Social Support							
Low	66/5536	ref		ref		ref	
Medium–High	128/11021	1.35 (0.64–2.84)	.43	1.40 (0.68–2.89)	.36	1.42 (0.69–2.95)	.34
Affection score							
Low	70/5624	ref		ref		ref	
Medium–High	124/10933	0.89 (0.55–1.45)	.65	0.87 (0.54–1.40)	.58	0.87 (0.54–1.40)	.56
Emotional & Info Support							
Low	68/5765	ref		ref		ref	
Medium–High	126/10792	1.38 (0.71–2.68)	.34	1.29 (0.67–2.48)	.45	1.28 (0.67–2.47)	.46
Tangible Social Support							
Low	76/5570	ref		ref		ref	
Medium–High	118/10987	0.67 (0.45–0.99)	.05	0.65 (0.43–0.98)	.04	0.65 (0.43–0.97)	.04
Positive Social Interaction							
Low	67/6082	ref		ref		ref	
Medium–High	127/10475	1.58 (0.98–2.54)	.06	1.54 (0.95–2.51)	.08	1.54 (0.95–2.50)	.08
Satisfaction with life							
Low	80/5855	ref		ref		ref	
Medium–High	114/10702	1.37 (0.85–2.22)	.20	1.49 (0.92–2.41)	.11	1.48 (0.91–2.40)	.11
Log of social participation	194/16557	0.83 (0.69–1.01)	.07	0.87 (0.72–1.06)	.17	0.89 (0.73–1.07)	.21
Loneliness							
Rarely/never (< 1 day/week)	137/12751	ref		ref		ref	
Felt lonely (≥ 1 day/week)	57/3806	1.49 (0.90–2.45)	.12	1.60 (0.99–2.59)	.06	1.58 (0.97–2.56)	.06
Depression							
No	152/14411	ref		ref		ref	
Yes	42/2146	2.00 (1.21–3.32)	.01	1.68 (1.07–2.64)	.03	1.68 (1.07–2.65)	.02
Anxiety							
No	171/15256	ref		ref		ref	
Yes	23/1301	1.51 (0.83–2.74)	.18	1.40 (0.77–2.58)	.27	1.40 (0.77–2.57)	.27
PTSD							
No	173/15849	ref		ref		ref	
Yes	21/708	3.05 (1.70–5.49)	<.001	3.11 (1.71–5.66)	<.001	3.14 (1.74–5.67)	<.001
Log of network size	194/16557	0.93 (0.74–1.18)	.54	0.94 (0.74–1.19)	.61	0.95 (0.75–1.20)	.65

*CI* confidence interval, *OR* odds ratio, *PSEFs* psycho-socio-economic factors, *PTSD* post-traumatic stress disorder, ref denotes the reference category used for comparison

Model 1: Age group and sex

Model 2: Model 1 plus location, immigration status, BMI, total cholesterol, HDL, smoking, alcohol consumption, hypertension, diabetes diagnosis

Model 3: Model 2 plus physical activity

ORs for the log of social participation and the log of network size are per 1 standard deviation increase

Analytic weights from the second follow-up are used in the calculation of ORs and confidence intervals

## Discussion

In this large prospective cohort of over 16,000 Canadian adults, we found that CRM developed in 1.2% of participants over a follow-up range of 3–11 years, with incidence rates rising substantially with age and higher in men, urban residents and immigrants. Individuals who developed CRM were more likely to have adverse psycho-socio-economic profiles, including lower income and education, unemployment, and poor mental health. Notably, higher tangible social support was independently associated with reduced odds of CRM, while depression and PTSD were linked to significantly increased risk, underscoring the importance of psychosocial factors in the development of CRM beyond traditional socioeconomic markers.

Several studies have examined the associations between PSEFs and individual chronic conditions such as CVD and CKD. In a large meta-analysis involving over 26 million participants, Baruwa et al. [37] reported significantly higher CVD risk among individuals with low versus high income, and similarly for those with lower versus higher levels of education. Valtorta et al. [16] also showed that poor social relationships were associated with a greater risk of coronary heart disease and stroke, emphasizing the importance of social connectedness for cardiovascular health. Regarding CKD, Zeng and colleagues [15] demonstrated through a meta-analysis of 43 studies that lower income was consistently associated with increased CKD prevalence and progression, while lower educational attainment was linked to increased CKD prevalence. Although these findings underscore the importance of individual PSEFs in relation to CVD and CKD, no prior studies have prospectively examined their associations with the combined endpoint of CRM. Our group has recently reported cross-sectional associations between PSEFs and CRM using baseline data from the same cohort, where we found that CRM prevalence increased with age and was higher in men and rural residents [8]. In that analysis, homeownership and moderate household income were each associated with lower odds of CRM, but other PSEFs showed no clear associations [8]. In contrast, the current prospective analysis reveals a stronger role for psychosocial factors, particularly tangible social support, depression, and PTSD, in influencing CRM risk, suggesting that these dimensions may play a more critical role in the development, rather than merely the presence, of CRM over time. These differences highlight the value of longitudinal assessments in uncovering dynamic risk factors that may not be evident in cross-sectional analyses.

The observed associations between PSEFs and the development of CRM are likely underpinned by a complex interplay of behavioral, psychosocial, and structural mechanisms. Individuals with lower income, lower education, unemployment, and poor mental health may experience multiple disadvantages that negatively impact their health [38, 39]. These include greater exposure to chronic stress, reduced participation in health-promoting behaviors, inadequate diets, physical inactivity, and lower engagement with preventive healthcare services [40–42]. These behavioral and systemic factors can, in turn, increase the risk of obesity, diabetes mellitus, and hypertension, well-established precursors to both CVD and CKD. Educational attainment plays a pivotal role in shaping health outcomes. Those with lower education may have limited health literacy, leading to poorer decision-making and reduced access to effective healthcare interventions [43]. In contrast, higher educational levels often correlate with greater health knowledge, enabling better navigation of the healthcare system and adoption of protective behaviors [44]. Importantly, this study identified higher tangible social support as a protective factor against CRM. The protective effects of social support on cardiometabolic and renal health are mediated through several interrelated biopsychosocial pathways. These include modulation of the hypothalamic–pituitary–adrenal (HPA) axis, leading to reduced stress reactivity and cortisol secretion; enhanced parasympathetic activity and vagal tone; lower levels of systemic inflammation and improved immune competence; and psychological benefits such as improved coping, reduced depressive symptoms, and enhanced self-efficacy [45, 46]. Social support also promotes health-enhancing behaviors, including greater adherence to medication and treatment regimens, increased physical activity, healthier dietary patterns, better sleep hygiene, and timely healthcare utilization [47, 48]. Collectively, these pathways contribute to improved metabolic control, reduced progression of atherosclerosis, and ultimately lower risk of cardiovascular and renal complications. Conversely, poor mental health conditions such as depression and PTSD may exacerbate physiological stress responses, reduce motivation to engage in healthy behaviors, and impair disease self-management, [49] thereby increasing CRM risk. Our findings also revealed that CRM incidence was higher among men compared to women, urban compared to rural residents, and immigrants compared to non-immigrants. These differences likely reflect sex-based disparities in health behaviors, biological susceptibilities, and healthcare utilization patterns, with men typically engaging less in preventive care and exhibiting higher risk factor burden [50, 51]. Urban residents may experience greater environmental and lifestyle stressors, including pollution, sedentary behavior, and limited access to green spaces, that exacerbate chronic disease risk [52, 53]. Meanwhile, immigrants may face systemic barriers to care, language and cultural differences, and socioeconomic disadvantages that impair chronic disease prevention and management [54, 55]. These structural and contextual exposures may interact with PSEFs to heighten CRM vulnerability in these subgroups.

These findings underscore the critical role of psychosocial factors, particularly tangible social support, depression, and PTSD, in the development of CRM among middle-aged and older adults. Clinically, they suggest the need for routine screening of mental health conditions and assessment of social support as part of chronic disease prevention strategies in primary care. From a public health perspective, interventions aimed at enhancing social connectedness, improving access to mental health services, and targeting high-risk groups, such as older adults, men, urban residents, and immigrants, may help mitigate the growing burden of CRM. These insights also reinforce the importance of integrating psychosocial dimensions into multimorbidity care models and policies aimed at healthy aging.

This study has several notable strengths. It utilized data from a large, nationally representative cohort of middle-aged and older adults, enhancing the generalizability of the findings to the Canadian population. The prospective design, along with the exclusion of individuals with CRM at baseline, enabled the temporal assessment of PSEFs and incident CRM. Furthermore, the use of the CLSA Comprehensive Cohort allowed for detailed measurement of health, behavioral, and psychosocial exposures, enabling comprehensive adjustment for a wide range of potential confounders. However, the study also has limitations. The observational nature of the design precludes causal inferences, and residual confounding from unmeasured or mismeasured variables cannot be ruled out. The absence of precise dates for CRM diagnoses limited time-to-event analyses. Another key limitation of our study is the lack of longitudinal data on the progression and severity of mental health conditions such as depression and PTSD. This restricts our ability to evaluate how temporal patterns (e.g., chronicity or fluctuation) or symptom severity may influence the risk of CRM development. Future studies incorporating repeated assessments of mental health and CRM onset are needed to better understand these dynamic relationships. Additionally, the CLSA sampling frame excluded certain populations, such as residents of the three territories, those living on First Nations reserves, institutionalized individuals, and full-time members of the Canadian Armed Forces, potentially leading to underestimation of CRM incidence. The reliance on self-reported disease status introduces a risk of misclassification, though previous research suggests moderate agreement with clinical records. records [56, 57]. Our approach to resolving inconsistencies in self-reported chronic conditions across waves may have introduced misclassification bias, potentially leading to a slight overestimation of CRM incidence. Lastly, although our study is among the first to prospectively examine psycho-socio-economic determinants of CRM, the relatively small number of CRM events may have limited statistical power to detect modest associations and precluded meaningful subgroup analyses by age, sex, geographical location, and immigration status. Future studies using larger cohorts or pooled analyses through individual participant data meta-analyses (e.g., combining CLSA with the English Longitudinal Study of Aging, ELSA) are warranted to strengthen inference and uncover potential heterogeneity in associations.

## Conclusions

In this large, nationally representative cohort of middle- to older-aged adults, the incidence of CRM was relatively low but increased markedly with age and was higher among men, urban residents and immigrants. Among the PSEF factors examined, higher tangible social support was independently associated with a lower risk of developing CRM, whereas depression and PTSD were each linked to a higher risk. These findings emphasize the importance of psychosocial well-being in the prevention of interconnected chronic diseases such as cardiovascular and kidney disease. Interventions that enhance social connectedness and mental health support may play a meaningful role in reducing the burden of CRM. Further research in diverse populations is warranted to further explore these relationships and to inform policies and programs aimed at addressing psycho-socio-economic disparities in multimorbidity.

## Supplementary Information

Below is the link to the electronic supplementary material.

Supplementary file1 (DOCX 21 KB)

## Data Availability

Data are available from the Canadian Longitudinal Study on Aging (www.clsa-elcv.ca) for researchers who meet the criteria for access to de-identified CLSA data.
